# Supplementary material for: Targeting the interaction between RNA-binding protein HuR and FOXQ1 suppresses breast cancer invasion and metastasis
Source: Commun Biol. 2020 Apr 24;3:193. doi: 10.1038/s42003-020-0933-1 (PMC7181695; doi:10.1038/s42003-020-0933-1)
Supplement: Supplementary file 1 — Supplementary Information [file 42003_2020_933_MOESM1_ESM.pdf]

Supplementary figures

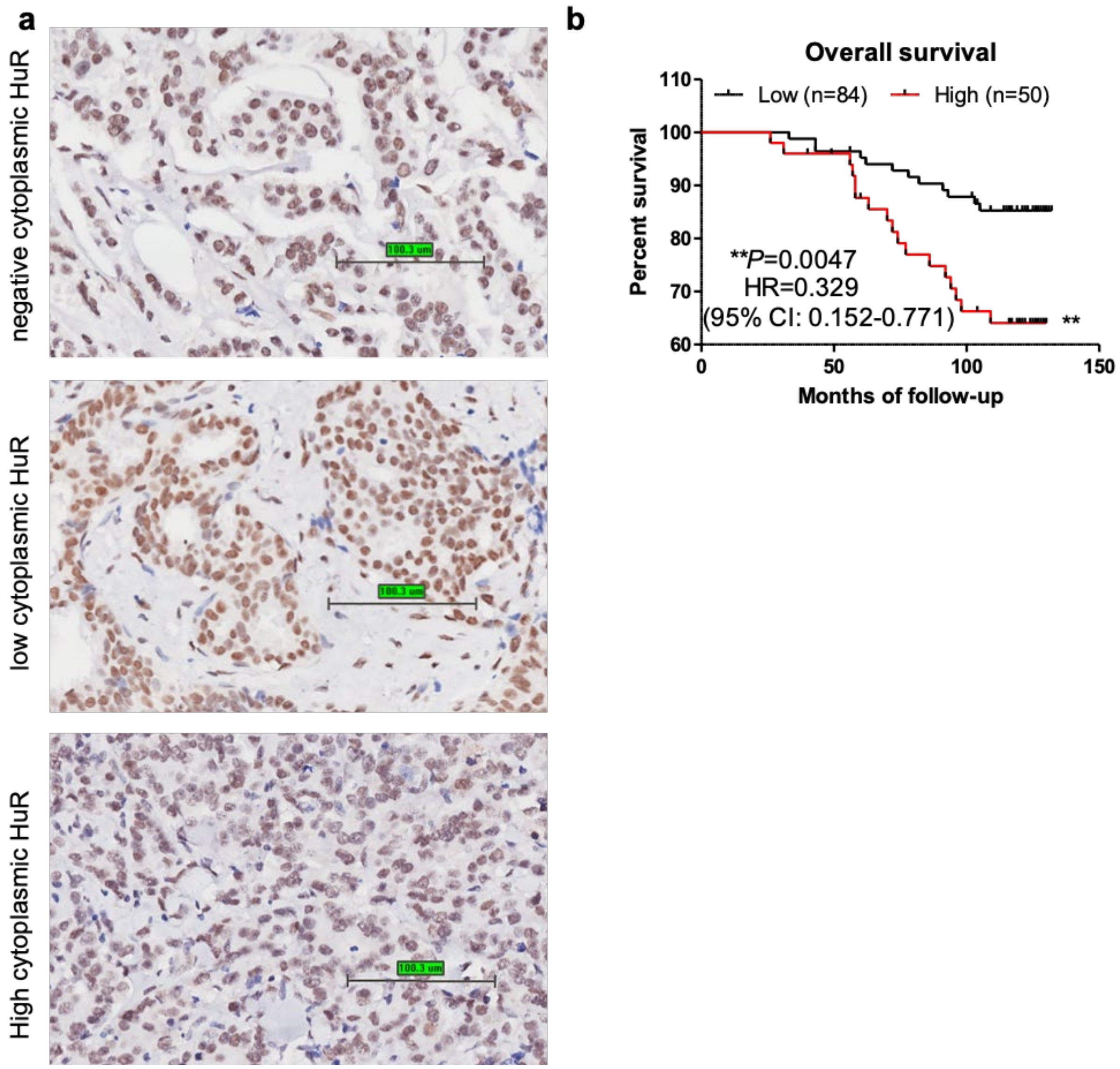

Supplementary Figure 1: a Representative images of HuR immunohistochemistry staining in breast cancer tissues, scale bars: 100.3 μm. **b** Kaplan-Meier analysis of the overall survival of 134 patients comparing high and low cytoplasmic HuR. Patients with high cytoplasmic HuR have lower overall survival rate compared to those with low cytoplasmic HuR (***P*=0.0047, log-rank test).

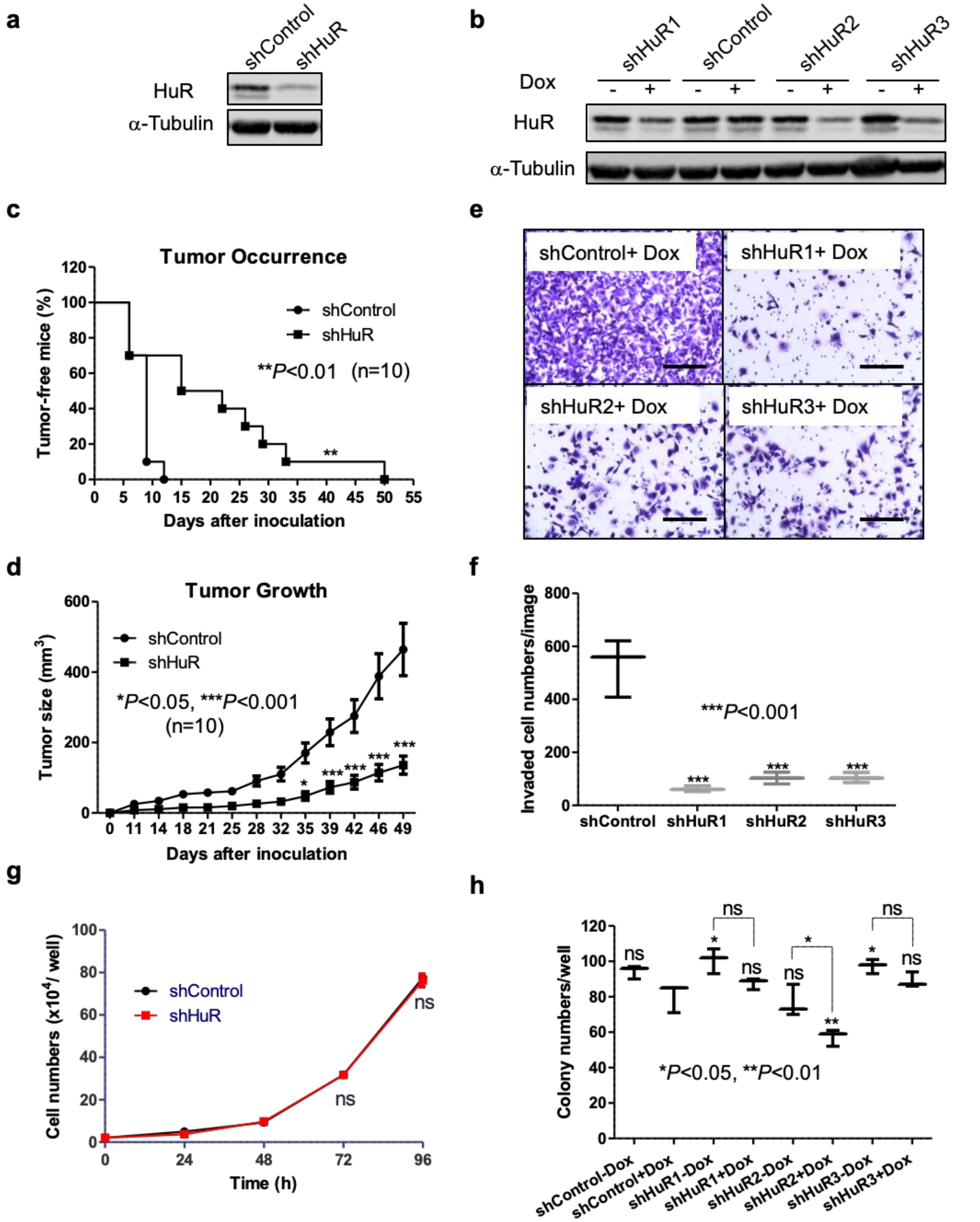

Supplementary Figure 2: HuR knockdown inhibits cell invasion and tumor formation. a-b HuR expression in MDA-MB-231 cells stably transduced lentiviral constant shRNA (**a**) or

doxycycline inducible shRNA (**b**). **c-d** Tumor initiation (**c**) and tumor growth (**d**) of MDA-MB-231 cells with HuR constant knockdown in a mammary fat pad xenograft model. HuR knockdown delayed tumor occurrence (n=10, $^{**}P<0.01$, log-rank test) and decreased tumor growth (n=10, $^{*}P<0.05$, $^{***}P<0.001$, two-way ANOVA). **e-f** Invasion assay in MDA-MB-231 cells with inducible HuR knockdown. **e** Representative images of stained invaded cells, scale bars: 200 μm. **f** The number of invaded cells per image ($^{***}P<0.001$, one-way ANOVA, n=4). **g** Growth curves of MDA-MB-231 cells with shControl or HuR constant knockdown. Values are mean ± SD from three independent experiments (ns: not significant, two-way ANOVA). **h** Colony formation of MDA-MB-231 cells with inducible HuR knockdown (ns: not significant, $^{*}P<0.05$, $^{**}P<0.01$, one-way ANOVA, n=3).

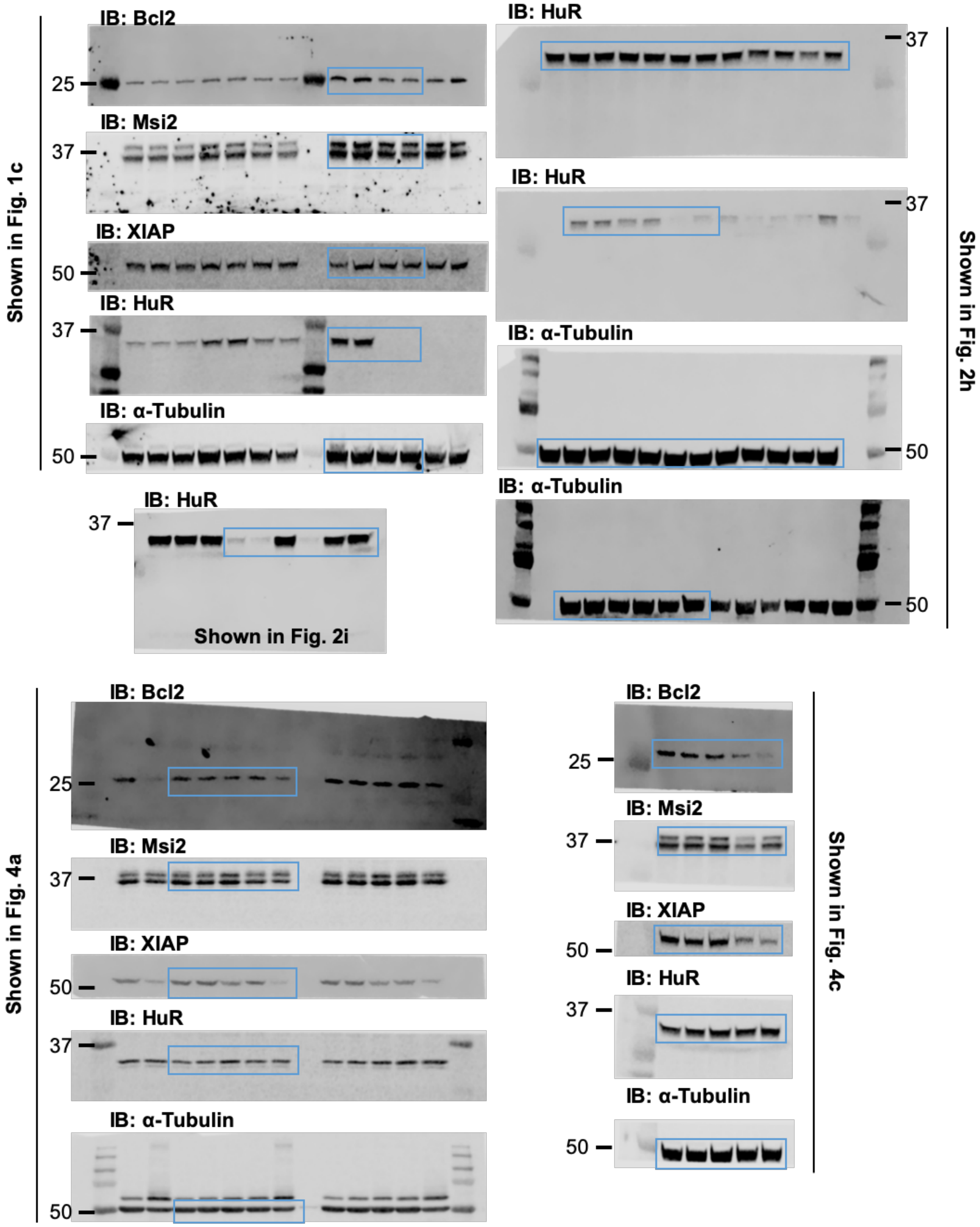
Shown in Fig. 1c
IB: Bcl2
25
IB: Msi2
37
IB: XIAP
50
IB: HuR
37
IB: α-Tubulin
50
IB: HuR
37
Shown in Fig. 2i
Shown in Fig. 2h
IB: HuR
37
IB: HuR
37
IB: α-Tubulin
50
IB: α-Tubulin
50
Shown in Fig. 4a
IB: Bcl2
25
IB: Msi2
37
IB: XIAP
50
IB: HuR
37
IB: α-Tubulin
50
Shown in Fig. 4c
IB: Bcl2
25
IB: Msi2
37
IB: XIAP
50
IB: HuR
37
IB: α-Tubulin
50

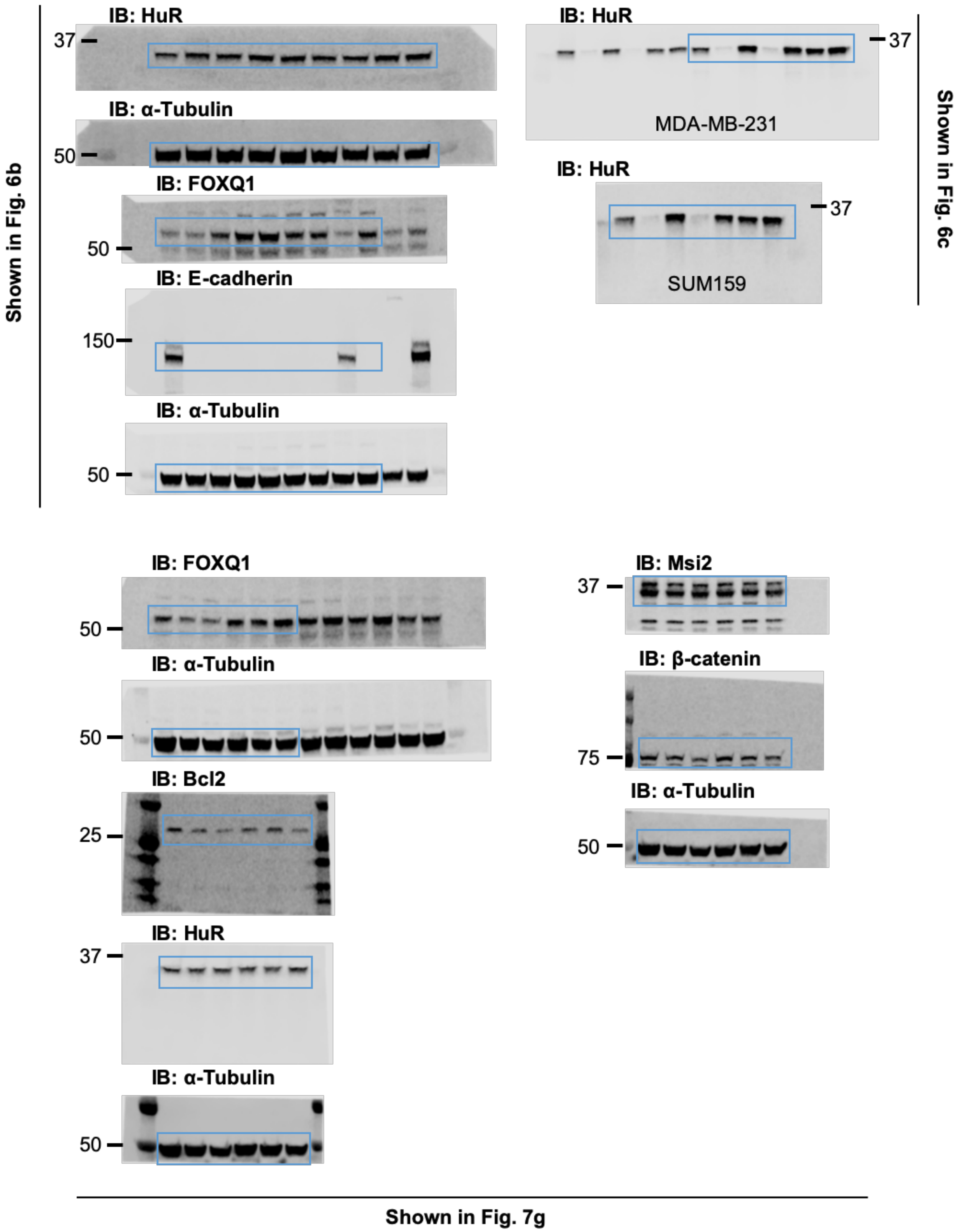

Shown in Fig. 6b
IB: HuR
37
IB: α-Tubulin
50
IB: FOXQ1
50
IB: E-cadherin
150
IB: α-Tubulin
50
IB: HuR
37
MDA-MB-231
IB: HuR
37
SUM159
Shown in Fig. 6c
IB: FOXQ1
50
IB: α-Tubulin
50
IB: Bcl2
25
IB: HuR
37
IB: α-Tubulin
50
IB: Msi2
37
IB: β-catenin
75
IB: α-Tubulin
50
Shown in Fig. 7g

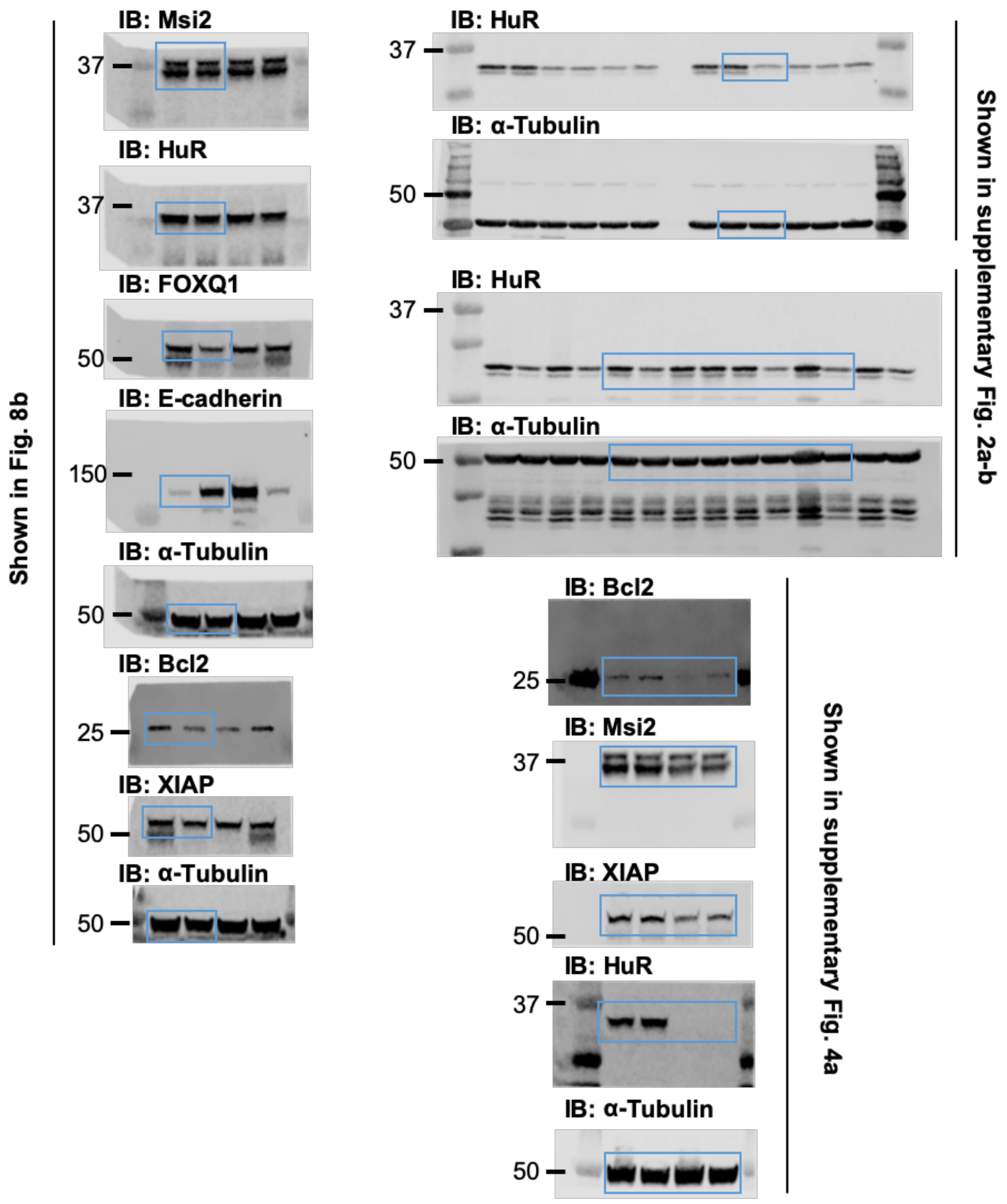

Supplementary Figure 3: Uncropped immunoblots for Fig. 1c, Fig. 2h-i, Fig. 4a, Fig. 4c, Fig. 6b-c, Fig. 7g, Fig. 8b, Supplementary Fig. 2a-b and Supplementary Fig. 4b.

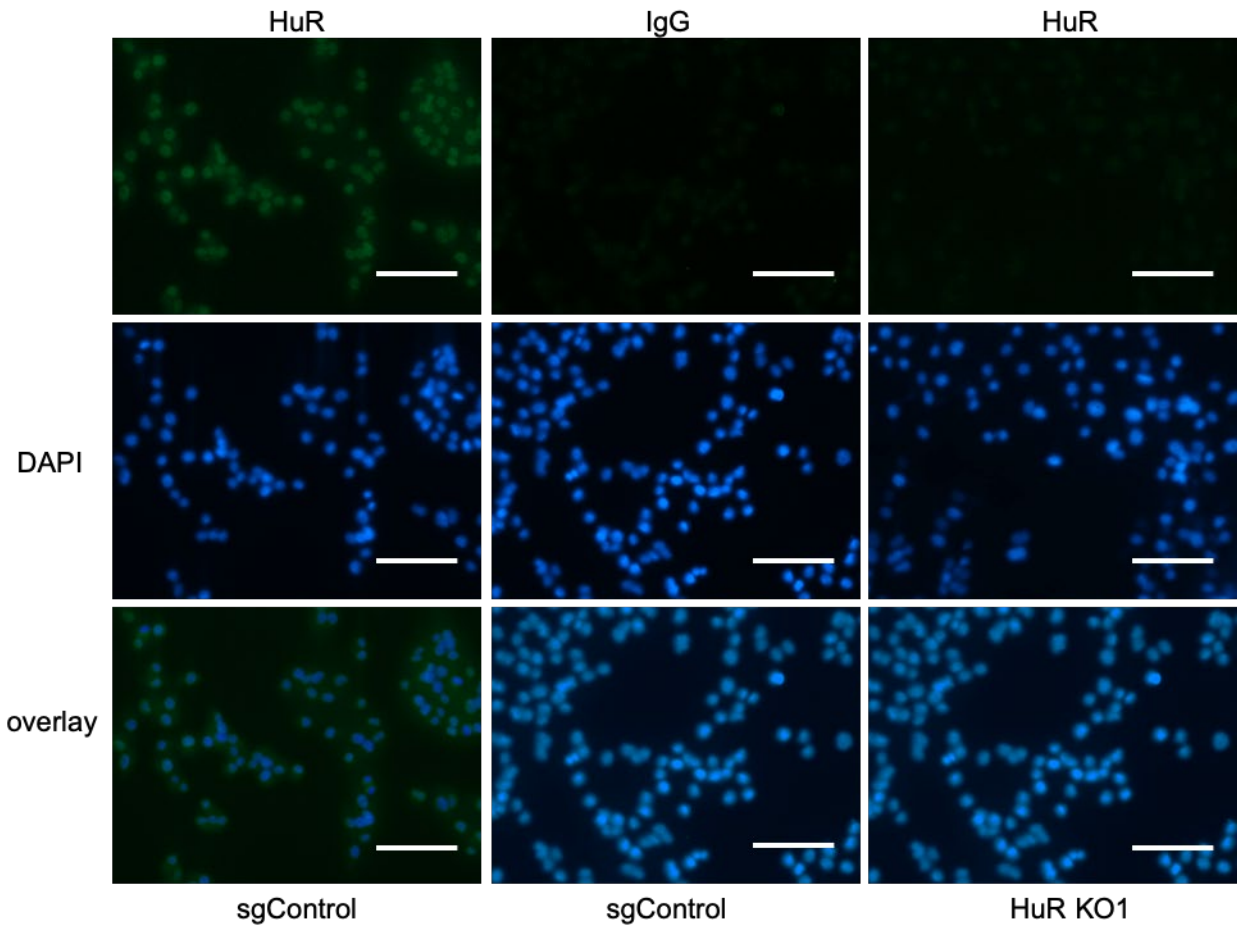

Supplementary Figure 4: Representative immunofluorescence images showing HuR (green) or nuclei (DAPI) in MDA-MB-231 sgControl cells and HuR KO1 cells, scale bars: 200 µm.

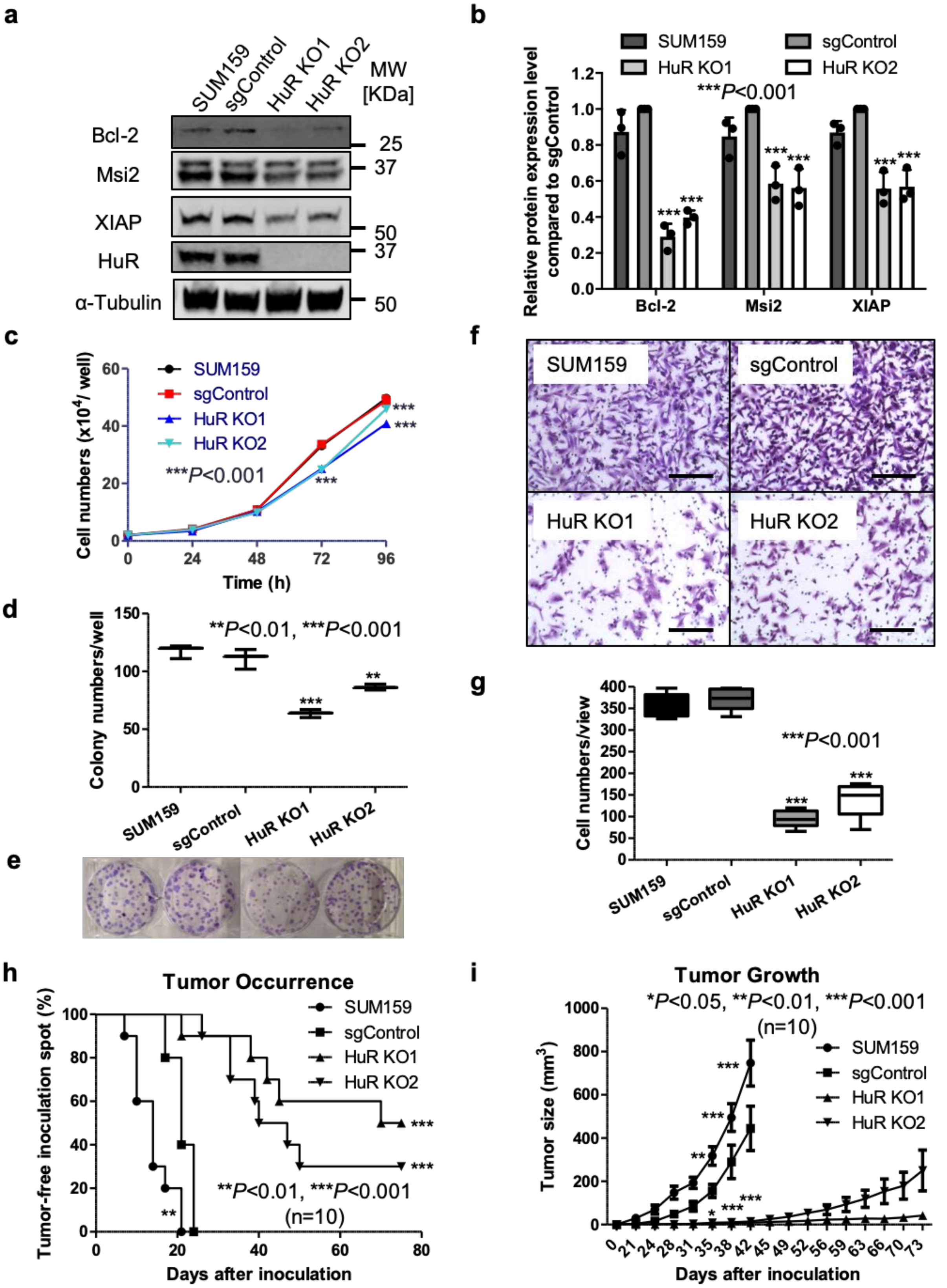
a
SUM159
sgControl
HuR KO1
HuR KO2
MW [KDa]
Bcl-2
Msi2
XIAP
HuR
α-Tubulin
25
37
50
37
50
b
SUM159
sgControl
HuR KO1
HuR KO2
***P<0.001
Relative protein expression level compared to sgControl
Bcl-2
Msi2
XIAP
c
SUM159
sgControl
HuR KO1
HuR KO2
***P<0.001
Cell numbers (x10⁴/ well)
Time (h)
d
**P<0.01, ***P<0.001
Colony numbers/well
SUM159
sgControl
HuR KO1
HuR KO2
e
f
SUM159
sgControl
HuR KO1
HuR KO2
g
***P<0.001
Cell numbers/view
SUM159
sgControl
HuR KO1
HuR KO2
h
Tumor Occurrence
SUM159
sgControl
HuR KO1
HuR KO2
**P<0.01, ***P<0.001
(n=10)
Tumor-free inoculation spot (%)
Days after inoculation
i
Tumor Growth
*P<0.05, **P<0.01, ***P<0.001
(n=10)
SUM159
sgControl
HuR KO1
HuR KO2
Tumor size (mm³)
Days after inoculation

Supplementary Figure 5: HuR knockout in SUM159 cells inhibits cell invasion and tumor formation. a-b Protein expression levels of HuR and downstream targets in SUM159 cells, cells with control sgRNA (sgControl) and two HuR KO clones. **a** Representative WB results from one experiment. **b** Quantified relative expression of HuR downstream target Bcl-2, Msi2 and XIAP. Values are mean ± SD from three independent experiments (***P<0.001, two-way ANOVA). **c** Growth curves of SUM159 cells, sgControl and two HuR KO clones (***P<0.001, two-way ANOVA, n=3). **d-e** Colony formation of SUM159 cells, sgControl and two HuR KO clones. **d** Colony numbers per well (***P<0.001, one-way ANOVA, n=3). **e** Representative images of colonies. **f-g** Invasion assay in SUM159 cells, sgControl and two HuR KO clones. **f** Representative images of stained invaded cells, scale bars: 200 μm. **g** The number of invaded cells per image (***P<0.001, one-way ANOVA, n=6). **h-i** Tumor initiation (**h**) and tumor growth (**i**)of SUM159 cells, sgControl and two HuR KO clones in athymic nude mice. Two HuR KO clones have delayed and less tumor formation as well as decreased tumor growth *in vivo* (n=10, ***P<0.001, log-rank test for tumor initiation and two-way ANOVA for tumor growth).

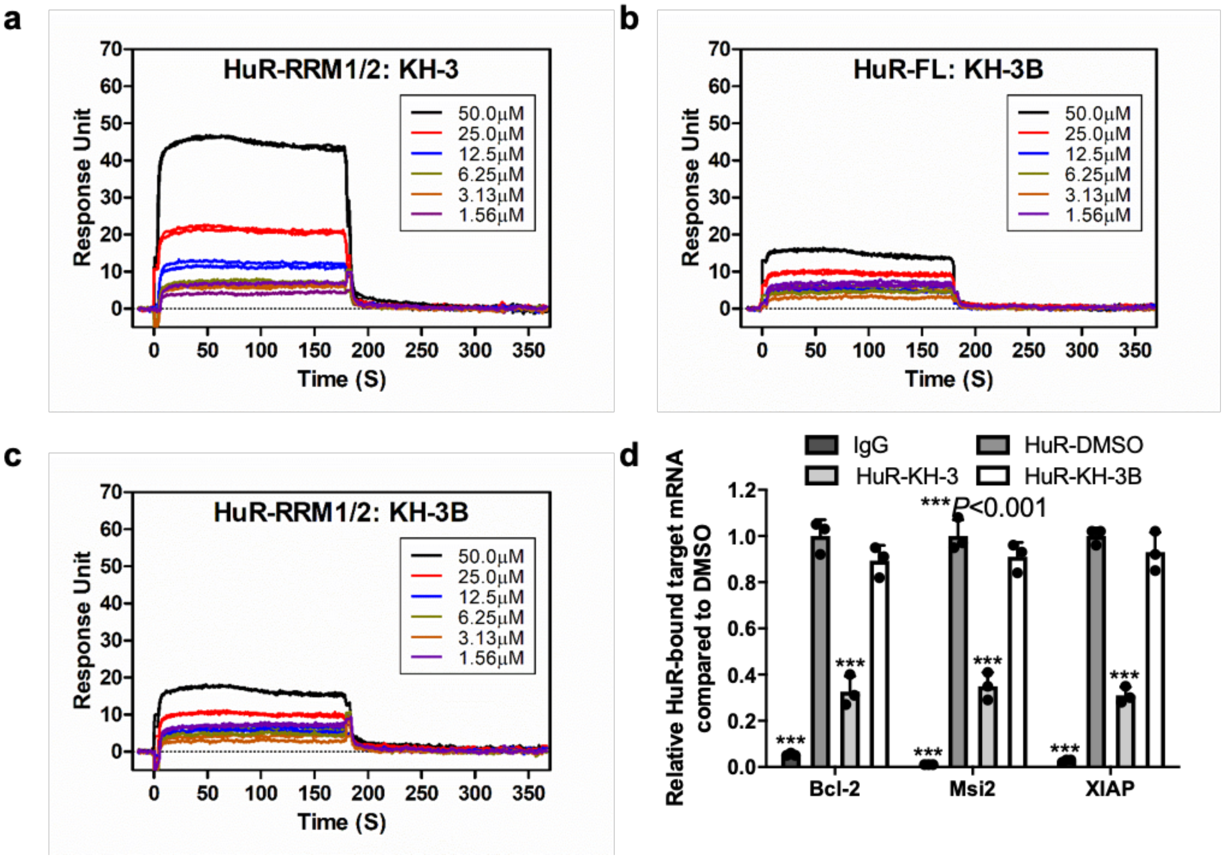

Supplementary Figure 6: a SPR analysis of KH-3 binding to immobilized HuR RRM1/2 protein. **b-c** SPR analysis of KH-3B binding to immobilized full-length HuR protein (**b**) and HuR RRM1/2 protein (**c**). Six doses were used and duplicated. **d** RNP IP analysis of HuR bound mRNA affected by KH-3 in SUM159 cells. Values are mean ± SD from three independent experiments (****P*<0.001, two-way ANOVA).

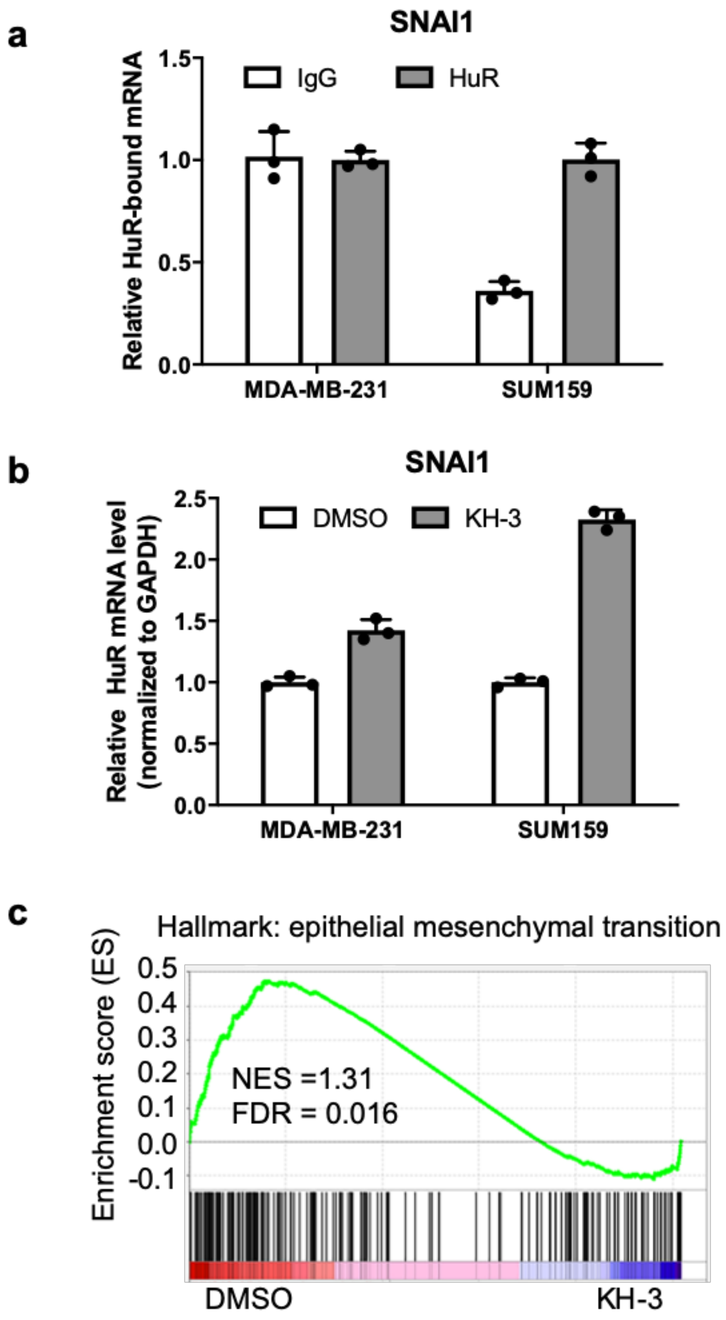

Supplementary Figure 7: a RNP IP analysis in MDA-MB-231 and SUM159 cells. HuR protein does not bind to SNAI1 mRNA in both cell lines. **b** SNAI1 mRNA expression in MDA-MB-231 and SUM159 cells with treatment of DMSO or 10 μM KH-3. KH-3 treatment increases SNAI1 mRNA expression. **c** Gene set enrichment analysis was used to analyze EMT gene set enrichment in DMSO and KH-3 treatment groups. Normalized enrichment score (NES) indicates the analysis

results across gene sets. False discovery rate (FDR) presents if a set was significantly enriched. ES, enrichment score.

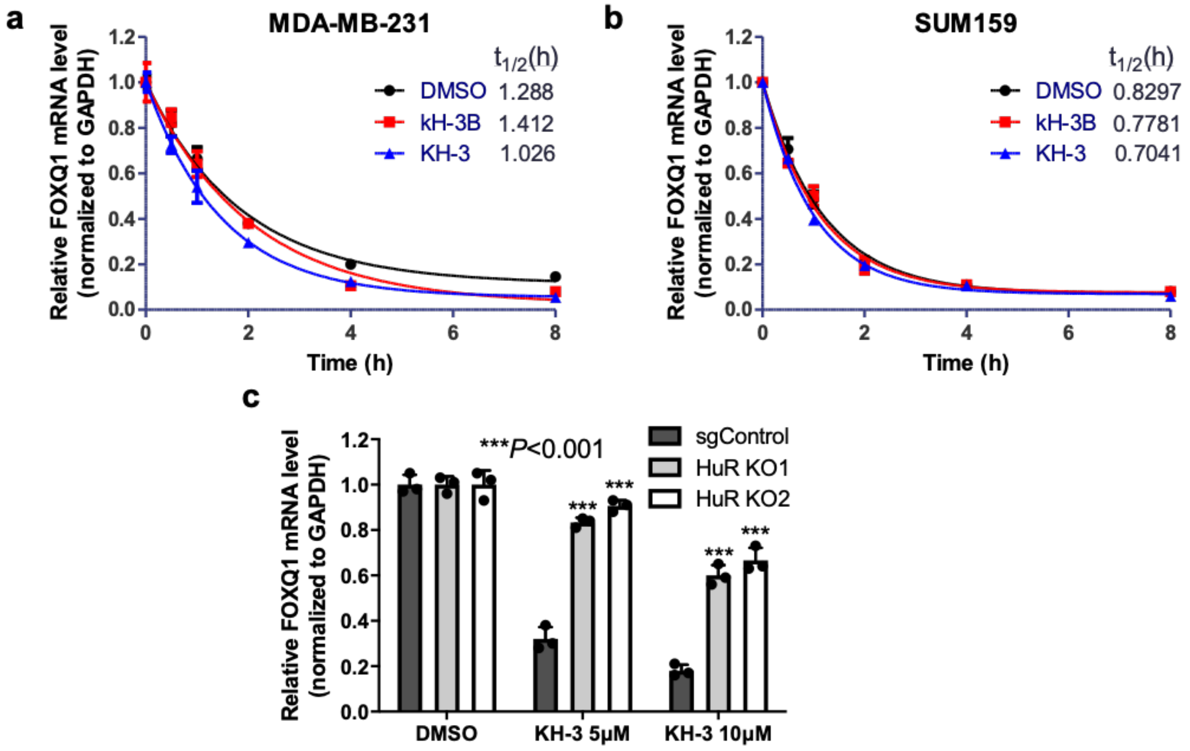

Supplementary Figure 8: a-b Half-life of FOXQ1 mRNA in MDA-MB-231 (**a**) and SUM159 (**b**) cells treated with 5 μg/mL actinomycin D together with DMSO, KH-3 or KH-3B. **c** FOXQ1 mRNA expression levels in MDA-MB-231 sgControl cells and two HuR KO clones treated with DMSO or KH-3. Values are mean ± SD from three independent experiments (****P*<0.001, two-way ANOVA).

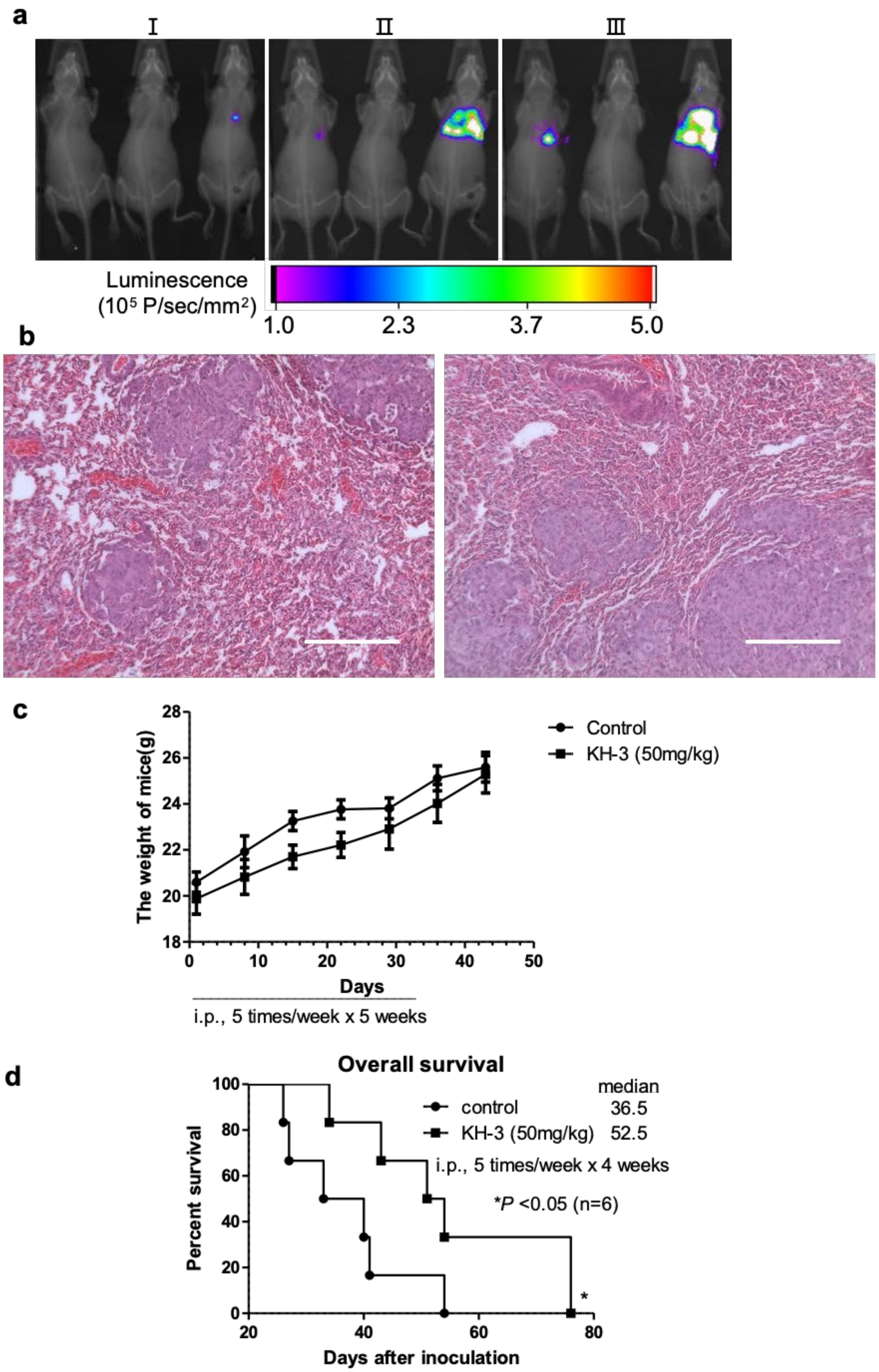

a
Ⅰ
Ⅱ
Ⅲ
Luminescence
(10^5 P/sec/mm^2)
1.0
2.3
3.7
5.0
b
c
The weight of mice(g)
Days
i.p., 5 times/week x 5 weeks
Control
KH-3 (50mg/kg)
d
Overall survival
Percent survival
Days after inoculation
median
control 36.5
KH-3 (50mg/kg) 52.5
i.p., 5 times/week x 4 weeks
*P <0.05 (n=6)

Supplementary Figure 9: a Representative bioluminescent images of same mice at three different stages of tumor progression. **b** Representative images of IHC H&E staining of lungs collected at the end of experiment. **c** Average body weight of mice receiving KH-3 or vehicle control treatment during the experimental duration. **d** Survival curves of mice in a 4T1 experimental metastatic model. KH-3 treatment improves survival time of mice (n=6, $^*P<0.05$, log-rank test).

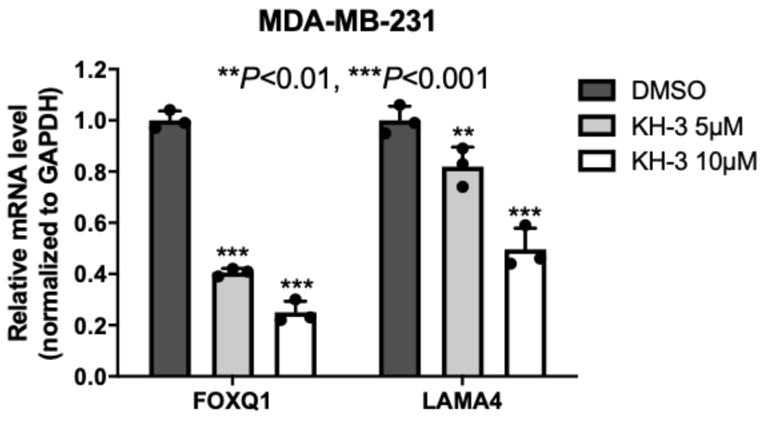

Supplementary Figure 10: FOXQ1 and LAMA4 mRNA expression in MDA-MB-231 cells with treatment of DMSO or KH-3. KH-3 inhibits the expression of both mRNAs in a dose-dependent manner. Values are mean ± SD from three independent experiments ($^{**}P<0.01$, $^{***}P<0.001$, two-way ANOVA).

Supplementary Table 1. Patients' clinicopathologic variables (n=140)

Parameter	No. patients (%)
Age, year	
Mean, Median (range)	55.9, 54 (29~87)
Tumor type	
IDC	128 (91.4%)
ILC	3 (2.1%)
Mucinous adeno	6 (4.3%)
others	3 (2.1%)
Tumor grade	
II	98 (70%)
II-III	37 (26.4%)
III	5 (3.6%)
Vascular invasion	
no	124 (88.6%)
yes	16 (11.4%)
T stage	
T1	57 (40.7%)
T2	81 (57.9%)
T3	2 (1.4%)
N stage	
N0	75 (53.6%)
N1	19 (13.6%)
N2	38 (27.1%)
N3	8 (5.7%)
AJCC clinical stage	
1	33 (23.6%)
2A-2B	61 (43.6%)
3A-3C	46 (32.8%)
Overall Survival, month *	
Mean, Median (range)	108.7, 121 (26~132)
live	110 (79.1%)
dead	29 (20.9%)
Relapse, month *	
Mean, Median (range)	100.5, 119 (10~132)
Yes/metastasis	38 (27.3%) /11 (7.9%)
No	101 (72.1%)

* n=139

Supplementary Table 2. Primer list for qPCR

Primers	Sequences
Bcl-2 forward	5′- CATGCTGGGGCCGTACAG -3′
Bcl-2 reverse	5′- GAACCGGCACCTGCACAC -3′
Msi2 forward	5′- CAACGACTCCCAGCACGAC -3′
Msi2 reverse	5′- GTCAATCGTCTTGGATCTAACTC -3′
XIAP forward	5′- AGTGGTAGTCCTGTTTCAGCATCA -3′
XIAP reverse	5′- CCGCACGGTATCTCCTTCA -3′
FOXQ1 forward	5′- CGAAGGAAGAGGGTACGACG -3′
FOXQ1 reverse	5′- GAGGGACGAACACCTCCAAC -3′
CDH1 forward	5′- CAGGAGTCATCAGTGTGGTCA -3′
CDH1 reverse	5′- GTTGTGCTTAACCCCTCACC -3′
CD82 forward	5′- GGCTGCTGAAGCAGGAGAT -3′
CD82 reverse	5′- TTGTAGAAGCTGACCCAGCC -3′
SNAI1 forward	5′- CTCTTTCCTCGTCAGGAAGC -3′
SNAI1 reverse	5′- GTCGTAGGGCTGCTGAAAG -3′
LAMA4 forward	5′- GACGCCAGGATAGCCAAGAA -3′
LAMA4 reverse	5′- ACAGCTTATGGTTGGGCAGT -3′
GAPDH forward	5′- ATGTTCGTCATGGGTGTGAA -3′
GAPDH reverse	5′- GGTGCTAAGCAGTTGGTGGT -3′
