# Supplementary material for: Targeting the interaction between RNA-binding protein HuR and FOXQ1 suppresses breast cancer invasion and metastasis
Source: Commun Biol. 2020 Apr 24;3:193. doi: 10.1038/s42003-020-0933-1 (PMC7181695; doi:10.1038/s42003-020-0933-1)
Supplement: Supplementary file 2 — Description of Additional Supplementary Files [file 42003_2020_933_MOESM2_ESM.pdf]

Description of Additional Supplementary Files

File Name: **Supplementary Data 1**
Description: RIP-seq analysis of HuR targets in MDA-MB-231 cells.

File Name: **Supplementary Data 2**
Description: RNA-seq analysis of differential expression in KH-3 vs DMSO treated MDA-MB-231 cells.

File Name: Supplementary Data 3
Description: Source data.
